# Multifocal hemorrhagic hepatocellular adenomas following vigorous physical exertion: Radiologic–pathologic correlation and endovascular management

**DOI:** 10.1016/j.radcr.2026.07.066

**Published:** 2026-08-15

**Authors:** Abdelkader Sqalli Houssaini, Ikram Sarsar, Chakir Mahfoud, Sara Cherkaoui, Ola Messaoud, Omar El Aoufir, Laila Jroundi, Zaynab Iraqi Houssaini

**Affiliations:** Emergency Radiology Department, Faculty of Medicine and Pharmacy, CHU Ibn Sina, University Mohammed V, Rabat, Morocco

**Keywords:** Hepatic adenoma, Inflammatory subtype, Spontaneous rupture, Hepatic hemorrhage, Steatosis, Transarterial embolization

## Abstract

We report a 41-year-old woman with long-term oral contraceptive use who developed acute right upper quadrant pain immediately after vigorous physical exertion. CT and MRI demonstrated multifocal hemorrhagic hepatocellular adenomas, including 12-cm and 3-cm lesions, both containing intralesional hemorrhage. The larger lesion was additionally associated with a subcapsular hematoma, without definite active contrast extravasation on CT. Persistent hemoglobin decline and worsening pain prompted selective arterial embolization, resulting in clinical and biological stabilization. Histopathological examination confirmed an inflammatory hepatocellular adenoma, and follow-up imaging demonstrated hematoma resolution and lesion regression. This case highlights that hemorrhagic changes may also occur in smaller adenomas and that the absence of visible active extravasation should not outweigh progressive clinical and laboratory evidence of ongoing bleeding when considering endovascular treatment.

## Introduction

Hepatocellular adenomas are uncommon benign liver tumors occurring predominantly in women of reproductive age and are associated with exposure to exogenous estrogens and metabolic risk factors. Their main complications are hemorrhage and, less frequently, malignant transformation. The risk of hemorrhage is influenced by lesion size and molecular subtype [[1], [2], [3]].

Multimodality imaging plays a central role in detecting intralesional hemorrhage and associated hematomas, demonstrating lesion multiplicity, assessing for active contrast extravasation, and guiding therapeutic management. In hemodynamically stable patients with hemorrhagic hepatocellular adenomas, selective arterial embolization may achieve bleeding control while preserving uninvolved liver parenchyma [4].

We present a case of multifocal hemorrhagic hepatocellular adenomas occurring immediately after vigorous physical exertion, illustrating the role of clinical, laboratory, and imaging findings in guiding selective arterial embolization despite the absence of definite active contrast extravasation on CT.

## Case report

A 41-year-old woman with long-term oral contraceptive use and no significant medical or surgical history presented with abrupt right upper quadrant pain immediately after vigorous physical exertion. She denied fever or jaundice. On admission, she was hemodynamically stable. Abdominal examination revealed right upper quadrant tenderness without signs of peritoneal irritation.

Initial laboratory tests showed anemia, with a hemoglobin level of 7.7 g/dL, and mild elevation of liver enzymes (AST, 62 U/L; ALT, 58 U/L).

Abdominal ultrasound demonstrated heterogeneous hepatic lesions containing internal echogenic components suggestive of hemorrhage, together with a small amount of perihepatic free fluid.

**Fig. 1:**
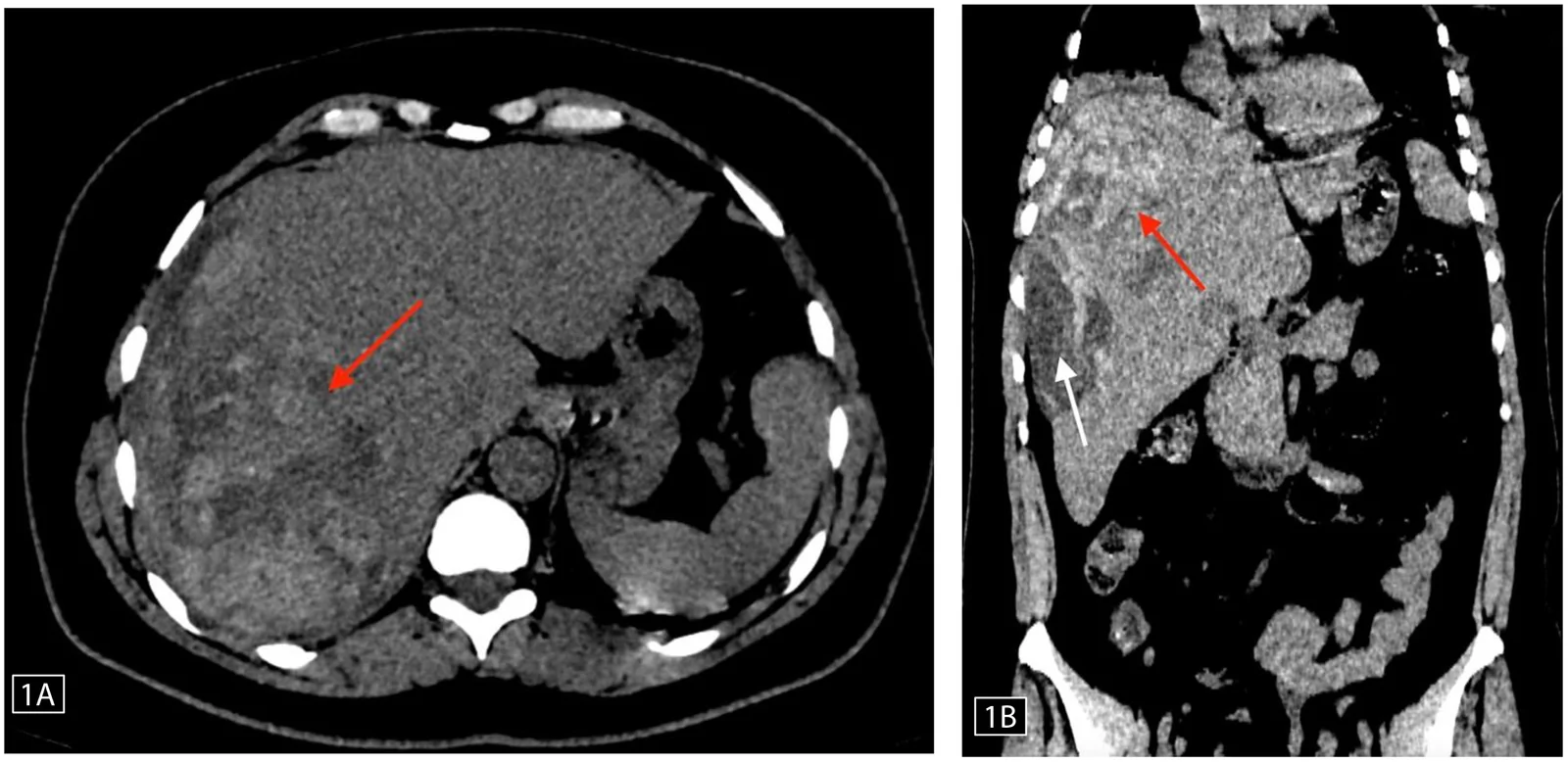
Unenhanced CT appearance of the largest hemorrhagic hepatocellular adenoma. (A and B) Axial and coronal unenhanced CT images showing a 12-cm heterogeneous, spontaneously hyperattenuating lesion involving segments VII and VIII, consistent with extensive intralesional hemorrhage (red arrow). The lesion has a subcapsular component with an associated subcapsular hematoma and causes focal bulging of the hepatic capsule (white arrow).

**Fig. 2:**
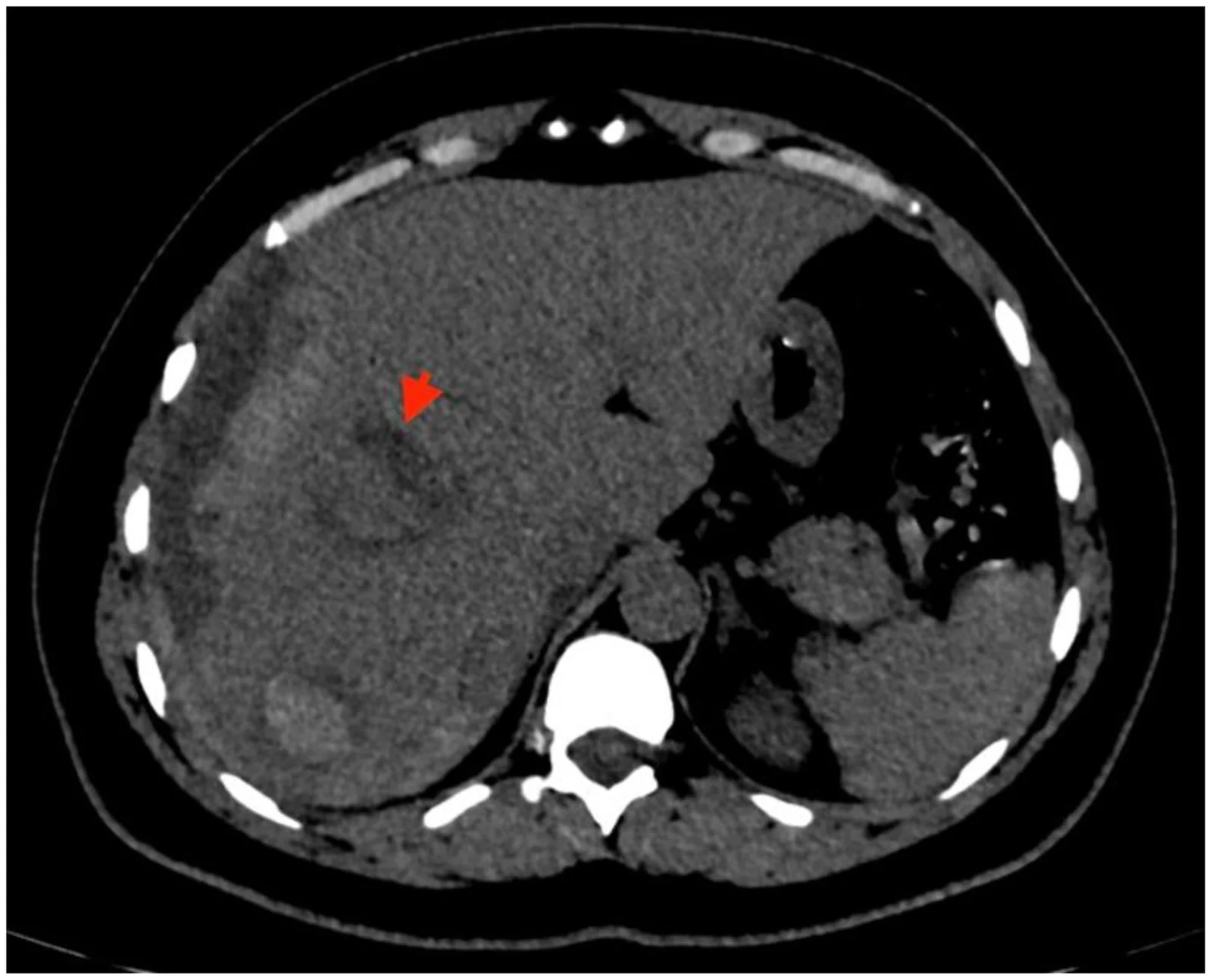
Unenhanced CT demonstrating a second distinct hemorrhagic hepatocellular adenoma in segment V. Axial unenhanced CT image showing a separate 3-cm lesion in hepatic segment V, containing spontaneously hyperattenuating areas consistent with intralesional hemorrhage (red arrowhead). This second distinct lesion confirms the multifocal nature of the hepatocellular adenomas.

Unenhanced CT demonstrated multiple distinct hemorrhagic hepatocellular adenomas. The 2 largest lesions were a 12-cm lesion involving segments VII and VIII (Fig. 1) and a separate 3-cm lesion in segment V (Fig. 2). Both lesions contained spontaneously hyperattenuating areas consistent with intralesional hemorrhage. The larger lesion extended to the subcapsular region and was associated with a subcapsular hematoma.

**Fig. 3:**
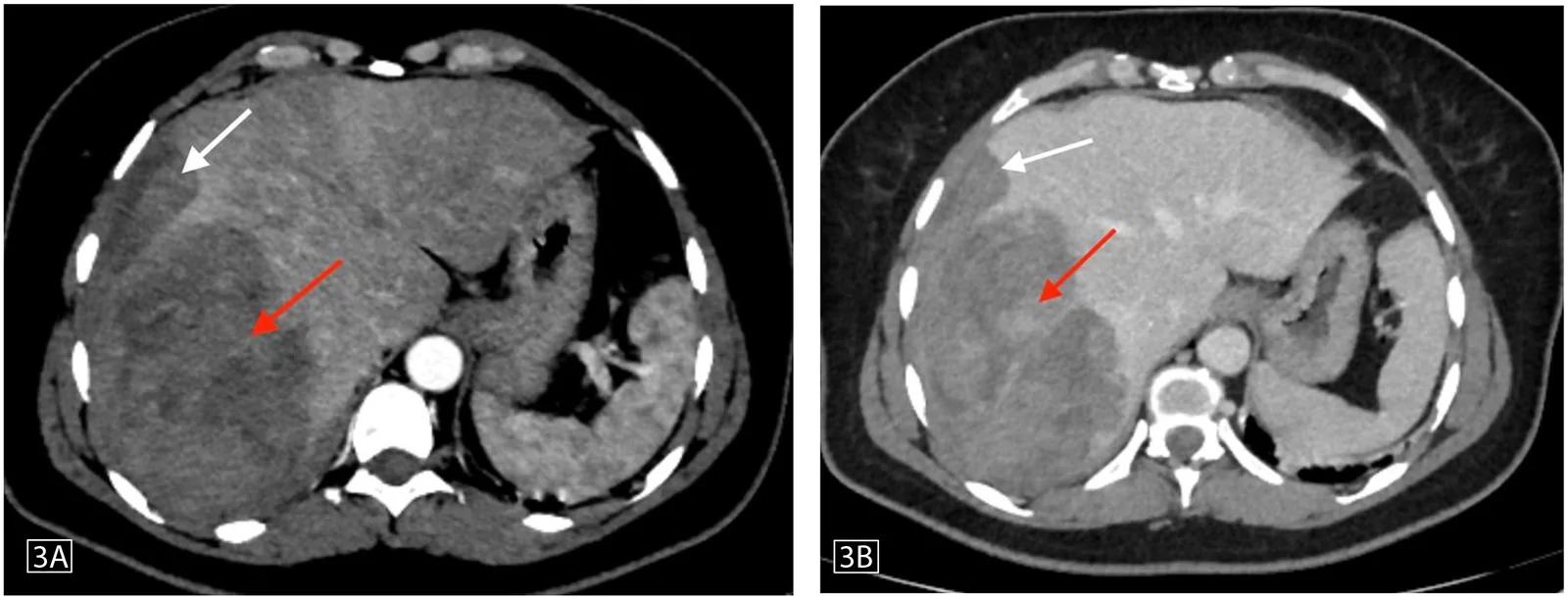
Contrast-enhanced CT appearance of the largest hemorrhagic hepatocellular adenoma. (A and B) Arterial and portal-phase contrast-enhanced CT images showing the large hemorrhagic lesion involving segments VII and VIII (red arrow) and the associated subcapsular hematoma (white arrow). The lesion and hematoma remain hypoattenuating relative to the surrounding liver parenchyma, without appreciable enhancement or definite active contrast extravasation.

On contrast-enhanced CT, the larger lesion and the associated subcapsular hematoma remained hypoattenuating relative to the surrounding liver parenchyma, without appreciable enhancement or definite active contrast extravasation (Fig. 3). No portal vein thrombosis or biliary dilatation was identified.

**Fig. 4:**
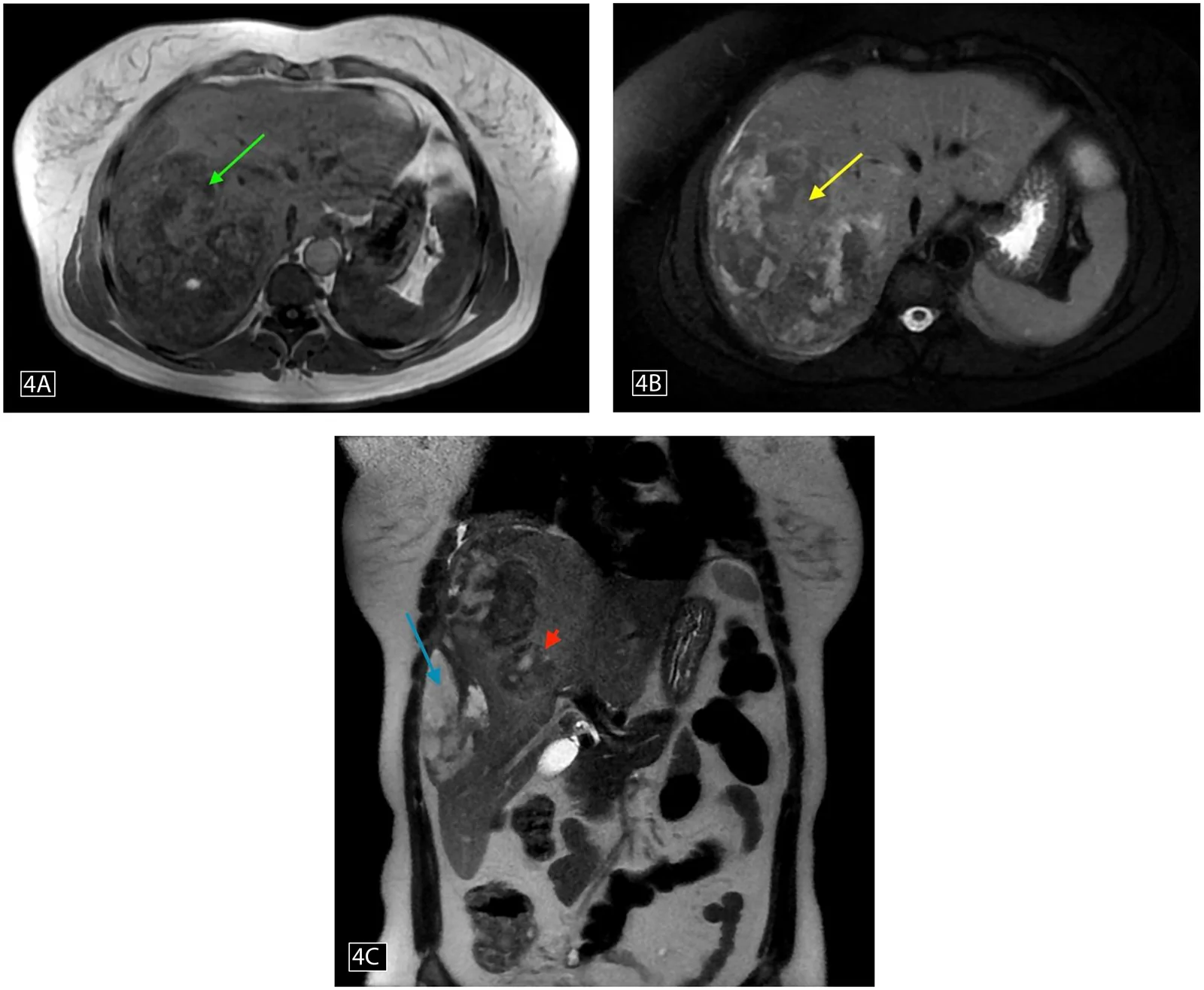
MRI appearance of multiple intrahepatic and subcapsular lesions. (A–C) MRI showing multiple intrahepatic lesions and an associated subcapsular hematoma. The largest lesion demonstrates heterogeneous predominantly isointense signal on T1-weighted imaging (green arrow) (A) and heterogeneous hyperintense signal on T2-weighted imaging (yellow arrow) (B). The coronal image (C) confirms its subcapsular component and demonstrates a separate smaller lesion in segment V (red arrowhead) with similar hemorrhagic imaging features, thereby confirming the multifocal nature of the disease.

**Fig. 5:**
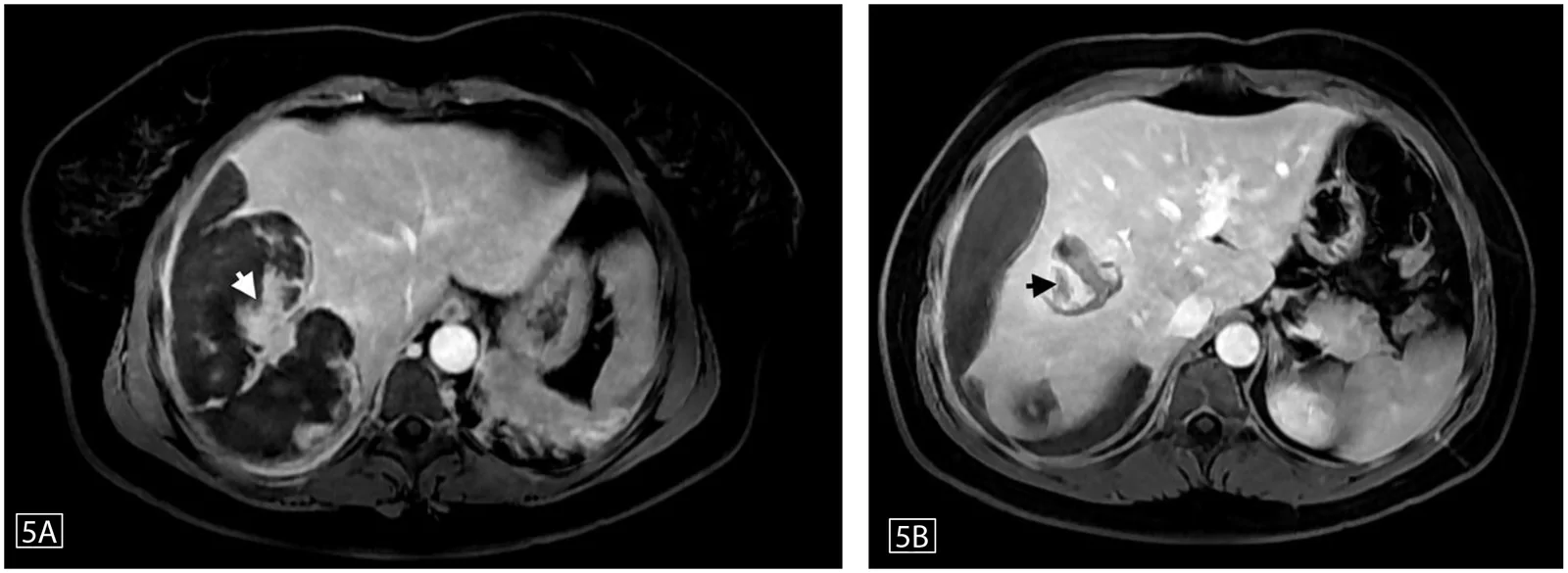
Dynamic MRI demonstrating arterial enhancement in 2 distinct hepatocellular adenomas. (A) Arterial-phase MRI showing heterogeneous enhancement within the viable components of the large hemorrhagic adenoma involving segments VII and VIII (white arrowhead). (B) Arterial-phase MRI demonstrating similar enhancement within the separate 3-cm adenoma located in segment V (black arrow).

MRI was subsequently performed and confirmed the presence of multiple hemorrhagic hepatocellular adenomas. The largest lesion demonstrated heterogeneous predominantly isointense signal on T1-weighted imaging and heterogeneous hyperintense signal on T2-weighted imaging, consistent with intralesional blood products. Coronal imaging confirmed its subcapsular component and associated hematoma and also demonstrated the separate 3-cm lesion in segment V with similar hemorrhagic imaging features, thereby confirming the multifocal nature of the disease (Fig. 4). On dynamic postcontrast MRI, viable portions of both the large lesion and the segment V lesion showed heterogeneous arterial enhancement (Fig. 5).

During clinical monitoring after completion of the imaging assessment, serial laboratory tests demonstrated a further decline in hemoglobin level, accompanied by worsening right upper quadrant pain. Given the ongoing clinical and biological suspicion of continued or intermittent bleeding, selective arterial embolization was performed despite the absence of definite active contrast extravasation on CT. The procedure resulted in clinical improvement and stabilization of the hemoglobin level.

**Fig. 6:**
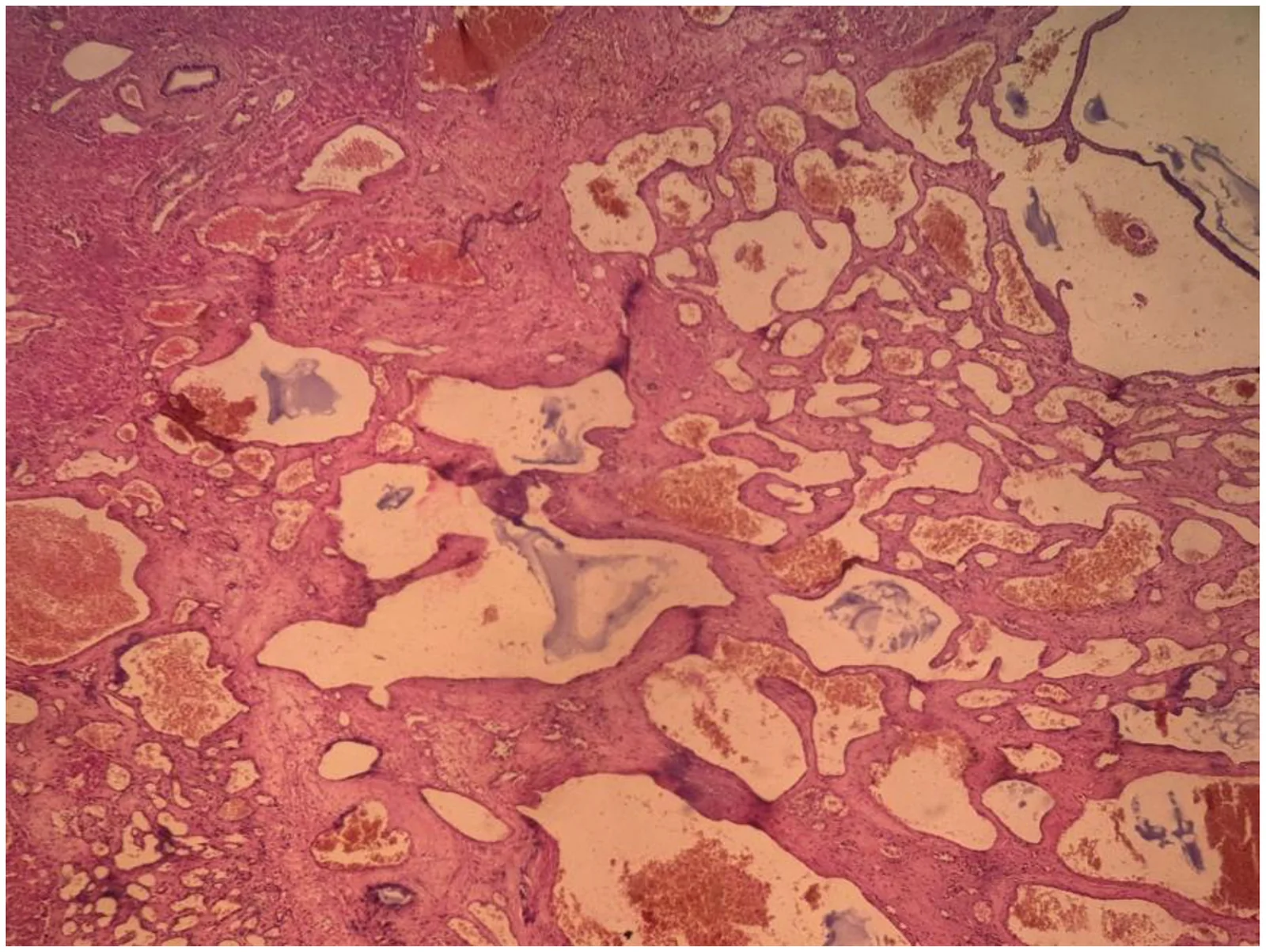
Histopathological features of ruptured inflammatory hepatic adenoma. Microscopic examination (H&E stain) shows dilated sinusoidal vascular channels with areas of intralesional hemorrhage and inflammatory stroma, consistent with an inflammatory subtype of hepatocellular adenoma complicated by rupture.

After selective arterial embolization and clinical and biological stabilization, tissue sampling of the larger lesion was performed. Histopathological examination confirmed an inflammatory hepatocellular adenoma with extensive hemorrhagic changes (Fig. 6).

The patient recovered uneventfully. At 3-month follow-up, CT showed regression of the embolized lesion and resolution of the subcapsular hematoma, with complete resolution of symptoms.

## Discussion

Hepatocellular adenomas are uncommon benign hepatocellular tumors occurring predominantly in women of reproductive age and are strongly associated with prolonged exposure to estrogen-containing oral contraceptives [1,2]. Their main complications are hemorrhage and malignant transformation. Hemorrhagic risk is influenced by lesion size and molecular subtype and is generally higher in lesions larger than 5 cm [2,3]. In the present case, however, intralesional hemorrhage was demonstrated in both the 12-cm lesion involving segments VII and VIII and the separate 3-cm lesion in segment V.

The current molecular classification includes HNF1A-inactivated, inflammatory, β-catenin–activated, mixed β-catenin–activated inflammatory, sonic hedgehog, and unclassified hepatocellular adenomas [2,3]. Inflammatory adenomas, which account for approximately 40% of cases, are characterized by sinusoidal dilatation, inflammatory infiltrates, and vascular ectasia and may have a marked hemorrhagic tendency [3,5]. Sonic hedgehog adenomas are also strongly associated with symptomatic bleeding, whereas HNF1A-inactivated adenomas generally have a lower hemorrhagic risk. β-catenin activation, particularly involving exon 3, is mainly associated with malignant transformation [2,3]. Histopathological examination confirmed the inflammatory subtype in the sampled lesion, but this classification cannot necessarily be extrapolated to all additional adenomas.

Multimodality imaging was essential for demonstrating lesion multiplicity and characterizing the hemorrhagic components. On unenhanced CT, the 2 largest lesions contained spontaneously hyperattenuating areas consistent with intralesional hemorrhage. The larger lesion was additionally associated with a subcapsular hematoma. On contrast-enhanced CT, the hemorrhagic components remained hypoattenuating relative to the surrounding liver parenchyma, without definite active contrast extravasation. MRI further characterized the blood products and demonstrated arterial enhancement within the viable portions of both lesions. Identification of the separate segment V adenoma on unenhanced CT, coronal MRI, and dynamic MRI confirmed the multifocal nature of the disease [1,2,5].

Mild hepatic steatosis was present but was not a predominant feature of this case. Although an association between hepatic steatosis and multiple hepatocellular adenomas has been reported [6,7], obesity and metabolic dysfunction were not documented in our patient. The onset of pain immediately after vigorous physical exertion represents a notable temporal association, although a direct causal relationship cannot be established.

Management of hemorrhagic hepatocellular adenomas depends on hemodynamic status, clinical progression, laboratory findings, and imaging features. Although the patient remained hemodynamically stable and no active contrast extravasation was identified on CT, persistent hemoglobin decline and worsening abdominal pain raised concern for ongoing or intermittent bleeding. Selective arterial embolization was therefore performed, resulting in clinical and biological stabilization while preserving uninvolved liver parenchyma. Follow-up imaging demonstrated regression of the subcapsular hematoma and reduction in lesion size, consistent with previously reported outcomes following transarterial embolization [4].

## Conclusion

Multifocal hepatocellular adenomas may be complicated by clinically significant intralesional hemorrhage and an associated subcapsular hematoma, even in the absence of definite active contrast extravasation on CT. In this setting, management should be guided by the integration of multimodality imaging with serial clinical and laboratory findings rather than by imaging evidence of active bleeding alone. Persistent hemoglobin decline and worsening abdominal pain may justify selective arterial embolization, which can achieve effective hemostasis while preserving uninvolved liver parenchyma. Careful assessment of all hepatic lesions is also essential, as hemorrhagic changes may involve smaller adenomas and help establish the multifocal nature of the disease.

## Ethics approval

Our institution does not require ethical approval for reporting individual cases.

## Patient consent

Written informed consent was obtained from the patients for their anonymized information to be published in this article.
